# Night-Time Biomass and Compositional Dynamics in *Chlorella vulgaris*: Optimisation of Harvesting Time

**DOI:** 10.3390/biotech15030064

**Published:** 2026-08-06

**Authors:** Sofia Pires, Susana Casal, Tânia G. Tavares, José C. M. Pires, Joana Oliveira

**Affiliations:** 1 LEPABE—Laboratory for Process Engineering, Environment, Biotechnology and Energy, ALiCE—Associate Laboratory in Chemical Engineering, Faculty of Engineering, University of Porto, Rua Dr. Roberto Frias, 4200-465 Porto, Portugal; up202108061@edu.fe.up.pt (S.P.); tsgtavares@fe.up.pt (T.G.T.); up201904660@edu.fe.up.pt (J.O.); 2 LAQV-REQUIMTE—Associated Laboratory for Green Chemistry of the Network of Chemistry and Technology, Faculty of Pharmacy, University of Porto, Rua de Jorge de Viterbo Ferreira 228, 4050-313 Porto, Portugal; sucasal@ff.up.pt

**Keywords:** biochemical composition, circadian cycle, microalgae, nocturnal variation

## Abstract

Global population growth has emphasised the need to have sustainable and alternative sources of nutrients. In this context, microalgae have emerged as a potential solution due to their rich biochemical composition, including high-quality proteins, carbohydrates, lipids, and pigments. This study investigates the variation in microalgal growth and biochemical composition over a light:dark cycle, with a focus on the night period. Batch experiments were performed with eight *Chlorella vulgaris* cultures over a 7-day period. On the seventh day, biomass samples were collected at four time points in four-hour intervals and stored for subsequent biochemical analyses. During the eight-hour dark period, biomass, carbohydrate, and total chlorophyll concentrations decreased by 9%, 12.5%, and 14.4%, respectively. After four hours of light exposure, these parameters increased significantly by 6%, 15.4%, and 12.7%, respectively. Total protein, carotenoids, and fatty acid contents remained relatively stable throughout the evaluated cycle, although variations were observed in the carotenoid profile. During the dark phase, zeaxanthin decreased by 38.0%, whereas violaxanthin increased by 27.2%, suggesting complementary pigment interconversion consistent with xanthophyll cycle activity. Overall, these results highlight the importance of optimising harvesting time to enhance the production of target compounds in a sustainable production of microalgal biomass.

## 1. Introduction

Microalgae are microscopic and mainly photosynthetic organisms that play a significant role in the primary food chain of aquatic ecosystems. They can rapidly grow in aquatic environments by efficiently using solar energy, a carbon source, and nutrients, producing approximately 50% of Earth’s total O_2_ through photosynthesis [1,2]. Microalgal growth rate, productivity, and biochemical composition are strongly influenced by various cultivation conditions, including cultivation temperature, nutrient availability, salinity, light intensity, photoperiod, light wavelength, and circadian cycle [3,4,5,6,7].

Among microalgal species, *Chlorella vulgaris* stands out as a well-established biotechnological reference organism, cultivated industrially for high-value bioproducts such as pigments (lutein, zeaxanthin), proteins, and polyunsaturated fatty acids, as well as for wastewater treatment and biofuel production [8,9]. Understanding how biomass composition fluctuates over a light:dark cycle is of direct relevance to process design: the timing of harvesting can substantially affect the yield and quality of target compounds, with variations in carbohydrate, pigment, and fatty acid content translating into measurable differences in product recovery and economic performance.

The circadian cycle lasts for 24 h, being regulated by an endogenous biological clock that influences physiological and behavioural processes in organisms, including microalgae. These cycles coordinate biological responses with environmental fluctuations and conditions such as light intensity and photoperiod, as well as temperature variations, synchronising these processes in the most suitable temporal window. During the light phase, microalgae use photosynthetic energy to fix carbon and accumulate intracellular reserves. In contrast, during the night period, UV- and light-sensitive processes (e.g., cell division and DNA replication) rely on these stored reserves. As a result, circadian rhythms are known to influence the biochemical composition of microalgae, particularly the concentrations of protein, carbohydrates, lipids, and pigments [4,5,6]. Some studies have reported similar behaviours in different microalgal species. For instance, while studying *Chlamydomonas* sp., Kato et al. [7] observed a decline in biomass concentration during the night phase, accompanied by decreases in lipid and carbohydrate contents. In another study, Sukenik and Carmeli [3] also reported reductions in carbohydrate and lipid contents in *Nannochloropsis gaditana*, along with decreases in protein, chlorophylls, and carotenoids during the night phase. Likewise, Fábregas et al. [5] observed decreases in lipid and protein contents under dark conditions; however, carbohydrate levels increased during this period. Conversely, Hidasi and Belay [6] reported increases in chlorophyll, carotenoid, and protein contents in *Arthrospira platensis* during the dark period, accompanied by a decrease in carbohydrate content. Similarly, Ogbonna and Tanaka [10] reported decreases in carbohydrate content and increases in protein content during the dark period in *Chlorella pyrenoidosa*. A different pattern was described by de Winter et al. [4] who noted that the variations in biochemical composition did not fully align with the light:dark cycle, with some experiments even showing cell division occurring during the light phase.

Although some studies have reported physiological adaptations of microalgae during the light:dark cycle, the novelty of this study lies in providing a time-resolved quantification of concurrent biomass, carbohydrates, proteins, fatty acids and pigments dynamics in *C. vulgaris* to establish a synchronised framework for precision industrial harvesting. Furthermore, the dynamics of biomass concentration and composition during the night phase remain insufficiently explored; therefore, the current study targets a focused analysis during that period. Thus, this work aims to map the variations in protein, carbohydrate, fatty acid, and pigments (including carotenoid profile) contents during a 16:8 h photoperiod, with particular emphasis on the dark phase, in *C. vulgaris*. The findings are discussed in the context of harvesting time optimisation and biomass valorisation, contributing to the rational design of production strategies for this industrially relevant species.

## 2. Materials and Methods

### 2.1. Microalgal Cultivation

*C. vulgaris* CCAP 211/11B was the selected strain for the current study, purchased from the Culture Collection of Algae and Protozoa (CCAP, Scotland, UK). This strain was previously inoculated in 100 mL Erlenmeyer flasks under continuous light with an intensity of 50 μmol m^−2^s^−1^, at room temperature, and with agitation at 120 rotations per min (rpm) provided by a Unimax 1010 orbital shaker (Heidolph, Schwabach, Germany). Low light intensity was used at this stage to avoid photoinhibition caused by low biomass concentration. After 30 days (with periodic sub-cultivation into fresh culture medium), the inoculum was scaled up to 2 L borosilicate flasks under the same photoperiod and a light intensity of 170 μmol m^−2^s^−1^. Aeration was provided at the bottom of the flasks (atmospheric air), which also provided agitation. In both stages, the inoculum was cultivated in modified OECD medium (Organisation for Economic Cooperation and Development), with the composition as detailed in Supplementary Materials (Table S1). The original composition of the OECD medium can be found in [11]. Prior to the experiments, the inoculum was harvested by centrifugation at 3745× *g* for 10 min at 4 °C, using a Gyrozen 1580R centrifuge (Gyrozen, Seoul, Republic of Korea).

Batch experiments with *C. vulgaris* were conducted over 7 days using eight round, clear 1 L borosilicate glass bottles with a starting biomass concentration of 151 ± 1 mg L^−1^ and the same modified OECD medium as used for inoculum cultivation. This initial biomass concentration was chosen to ensure sufficient final biomass amount for subsequent biochemical characterisation. A 16:8 h light photoperiod was used to grow the cultures, with a horizontal white-light LED panel positioned above the flasks. This photoperiod (16:8 h) and cultivation time (7 days) were previously selected by the research group as the most appropriate for the cultivation of the selected strain. The incident light intensity was 245.9 ± 16.4 μmol m^−2^s^−1^, measured on day 0 using a portable Delta OHM HD 2102.2 photo/radiometer (Delta OHM, Padua, Italy). To minimise potential heterogeneity in light supply, all flasks were placed equidistant from the light source and randomly repositioned daily. The cultivation temperature was 25 °C, kept using a Panasonic MLR-352 PE climate chamber. Continuous aeration was provided at a flow rate of 1.7 L min^−1^ using air pumps (Sicce Airlight 3300, Pozzoleone, Italy) equipped with sterile cellulose acetate 0.22 µm membrane filters. Aeration ensured proper homogenisation and facilitated the dispersion of atmospheric air (0.04% CO_2_) throughout the culture medium. Daily, the pH was adjusted to 7.0 ± 0.2 with H_2_SO_4_ or NaOH, and distilled water was added to compensate for evaporative water losses.

### 2.2. Biomass Harvesting

On the seventh day of growth, biomass from the eight vessels was harvested at four time points (biological duplicates) during the light:dark cycle, separated by four-hour intervals. The first two vessels were collected at the onset of the dark phase (Zeitgeber Time 0—ZT0), when the light was turned off. The second set was harvested four hours later, during the dark phase (ZT4); the third, when the new light phase started (ZT8); and the final set, four hours into the light period (ZT12). This sampling strategy enabled the evaluation of biomass concentration and composition dynamics across the dark phase, while the final time point captured transient, cyclic responses following the onset of light (ZT12).

At each sampling point, the entire volume of the bioreactors was harvested by centrifugation using a Gyrozen 1580R (Gyrozen, Seoul, Republic of Korea) at 3745× *g* (10 min; 4 °C). The obtained biomass was frozen at −80 °C for 24 h. The samples were then lyophilised, macerated, and stored under refrigeration for subsequent analyses.

### 2.3. Microalgal Growth Monitoring

Microalgal biomass concentration was monitored by measuring the optical density (OD) of the cultures at 680 nm on days 0, 1, 2, 3 and 6 of cultivation. Even though the use of a direct gravimetric method allows for a more reliable estimation of biomass concentration, OD correlations with biomass dry weight are widely reported in the literature and enable reduced sample volumes. Measurements were performed using a Shimadzu UV-1800 Spectrophotometer 240V IVDD (Shimadzu Corporation, Kyoto, Japan) with duplicate readings for all flasks. On day 7, OD was recorded every four hours to evaluate biomass variation throughout the twelve-hour evaluated period. OD values were converted to biomass concentrations (X, g L^−1^) according to Equation (1), using a previously established calibration curve as found in Supplementary Materials (Figure S1). For the exponential growth phase, the specific growth rate (μ, d^−1^) was calculated using Equation (2):

(1) $$\mathrm{OD}=(4.2\pm 0.1)\times (g\;L^{-1})+(0.05\pm 0.01),$$

(2) $$ln\frac{X_{2}}{X_{1}}=\mu (t_{2}-t_{1}),$$

where X_2_ and X_1_ represent biomass concentrations (g L^−1^) at final (t_2_) and initial (t_1_) times (d) of the exponential growth phase, respectively.

### 2.4. Biochemical Composition Analysis

#### 2.4.1. General Biochemical Composition Determination

Protocols for the determination of carbohydrate, protein and pigment contents followed the methodology of Esteves et al. [12], with chemical triplicates and results expressed as the weight percent of dry weight of algal biomass (% (wt)_DW_). The procedures for the determination of carbohydrates were based on the methods of DuBois et al. [13] and Van Wychen and Laurens [14]. In contrast, the methodologies for the determination of proteins and pigments were based on the methods of Lowry et al. [15] and Clément-Larosière et al. [16], respectively.

Pigment quantification

Pigments were extracted from 5 mg of biomass by homogenisation in 1 mL of 90% (*v*/*v*) methanol, followed by incubation in a water bath at 40 °C for 40 min. During incubation, samples were manually mixed every 10 min to improve the extraction efficiency. The resulting extracts were then centrifuged at 16,200× *g* for 5 min, and the supernatant was collected. To maximise pigment recovery, the extraction was performed a second time, and the supernatants obtained from both extraction steps were combined. Absorbance measurements of the pooled extracts were recorded at 652, 665, and 470 nm using a Spectroquant Prove 300 spectrophotometer (Merck, Darmstadt, Germany) equipped with a glass cuvette. The samples were diluted when necessary and, using Equations (3)–(6), which were retrieved from the literature [17], pigment concentrations were calculated as:

(3) $$C_{a}=16.82\times A_{665}-9.28\times A_{652},$$

(4) $$C_{b}=36.92\times A_{652}-16.54\times A_{665},$$

(5) $$C_{a+b}=0.28\times A_{665}+27.64\times A_{652},$$

(6) $$C_{car}=\frac{1000\times A_{470}-1.91\times C_{a}-95.15\times C_{b}}{225},$$

where C_a_, C_b_, C_a+b_, and C_car_ represent the concentrations (mg L^−1^) of chlorophyll a, chlorophyll b, total chlorophyll (a + b) and carotenoids, respectively, and A indicates absorbance at the corresponding wavelength.

Protein quantification

Protein extraction was carried out by suspending 5 mg of biomass in 4 mL of 1 N NaOH, followed by incubation in a water bath at 100 °C for 1 h. After cooling to room temperature, the samples were centrifuged at 1811× *g* for 30 min using an Eppendorf 5810 R centrifuge (Eppendorf, Hamburg, Germany). An aliquot of 500 µL of the resulting supernatant was then mixed with 5 mL of Lowry reagent and incubated in the dark for 10 min. Subsequently, 500 µL of 1 N Folin–Ciocalteu reagent was added, and the reaction mixture was maintained in the dark for a further 30 min to allow colour development. Absorbance was measured at 750 and 500 nm using a Spectroquant Prove 300 spectrophotometer (Merck, Darmstadt, Germany). Protein concentrations were determined using two calibration curves prepared with bovine serum albumin as the standard, as found in Supplementary Materials (Figure S2).

Carbohydrate quantification

Carbohydrates were extracted from 25 mg of lyophilised biomass by homogenisation with 0.25 mL of 72% (*w*/*w*) H_2_SO_4_, followed by incubation in a water bath at 30 °C for 1 h. Subsequently, 7 mL of ultrapure water was added, and the samples were hydrolysed in a thermoreactor at 120 °C for 1 h. After cooling to room temperature, the hydrolysates were centrifuged at 3220× *g* for 10 min using an Eppendorf 5810 R centrifuge (Eppendorf, Hamburg, Germany) to remove residual solids. For carbohydrate determination, 1 mL of the appropriately diluted supernatant was mixed with 1 mL of 5% (*w*/*v*) phenol solution and 5 mL of 95% (*w*/*w*) H_2_SO_4_. The reaction mixture was allowed to stand at room temperature for 10 min and was subsequently cooled in a cold-water bath for 15 min before further analysis. A Spectroquant Prove 300 spectrophotometer (Merck, Darmstadt, Germany) with a glass cuvette was then used to measure the absorbance at 490 nm. The total carbohydrate concentration was then calculated from a calibration curve previously generated with glucose standards, as shown in the Supplementary Materials (Figure S3).

To determine the percentage content of each component, the general equation shown in Equation (7) was used:

(7) $$Compound\;content\left(\%\right)=\frac{\left[Compound\right]\times V}{m_{BDW}}\times 100$$

where [Compound] represents the previously calculated concentration (mg L^−1^) of pigments, proteins and carbohydrates, V corresponds to the final volume (L) of sample extraction, and m_BDW_ is the accurate biomass dry weight used (mg).

#### 2.4.2. Carotenoid Profile Determination

Carotenoid profiling was performed following the method described by Pinto et al. [8] with slight modifications. Briefly, Precellys tubes were prepared with 150 mg of 0.1 mm glass beads and 25 mg of lyophilised biomass. Subsequently, 750 μL of acetone and 50 μL of trans-β-Apo-8′-carotenal solution (250 mg L^−1^; ExtraSynthese, Genay, France), used as an internal standard, were added to each tube. Cell disruption was carried out using a Precellys^®^ Evolution Touch homogeniser (Bertin Technologies, Montigny-le-Bretonneux, France). After cooling the samples on ice, two additional extraction cycles were performed: 750 μL of acetone was added to the Precellys tubes, and the homogenisation step was repeated to enhance carotenoid extraction. The extracts were then centrifuged at 3745× *g* for 10 min at 4 °C using a Gyrozen 1580R centrifuge (Gyrozen, Seoul, Republic of Korea). The recovered supernatants were filtered through 0.45 μm PTFE membrane filters and subsequently evaporated to dryness under a gentle nitrogen stream. The dried residues were reconstituted in a 9:1 (*v*/*v*) acetone:ethyl acetate solution prior to analysis. Carotenoids were identified and quantified by high-performance liquid chromatography (HPLC) according to the procedure reported by Pinto et al. [8]. Each condition had three independent samples analysed, and the results are expressed in milligrams per gram of dry algae (mg g_DW_^−1^).

#### 2.4.3. Fatty Acid Composition of Total Lipids

Fatty acid identification and quantification were performed according to the method described by Maia et al. [18], based on the protocol developed by Pagels et al. [19]. Briefly, 30 mg of biomass was supplemented with 50 μL of a 2 mg L^−1^ butylated hydroxytoluene (BHT) solution, used as an antioxidant, and 100 μL of a 2 mg L^−1^ triundecanoin solution as an internal standard. Subsequently, 2 mL of an acetyl chloride: methanol mixture (5:95, *v*/*v*) was added, and the samples were incubated at 80 °C for 1 h to promote the transesterification of fatty acids into their corresponding methyl esters. Following incubation, the reaction mixtures were allowed to cool to room temperature, after which 1 mL of 1% (*w*/*v*) NaCl solution and 1 mL of hexane were added to facilitate phase separation. The samples were then centrifuged at 3220× *g* for 5 min using a Heraeus Sepatech Labofuge AE centrifuge (Osterode, Germany). The organic phase containing the fatty acid methyl esters was recovered and dried over anhydrous sodium sulphate to remove residual moisture prior to chromatographic analysis. The clarified supernatant was used for injection into a gas chromatography with a flame ionisation detection (GC-FID) system, as described by Maia et al. [18]. For each condition, three independent analyses were conducted, with results expressed as the weight percent of dry weight of algal biomass (% (wt)_DW_).

### 2.5. Statistical Analysis

Technical triplicates were used for chemical analysis, while separate biological duplicates were used for cultivation experiments. The study’s evaluated parameters are presented as mean values with their associated standard deviations. JMP software (18.2.1, SAS Institute; Cary, NC, USA) was used to conduct statistical analyses. One-way analysis of variance (ANOVA) and Tukey’s Honest Significant Difference (HSD) test, with a statistical significance level of 0.05, were used to evaluate differences between samples.

## 3. Results and Discussion

### 3.1. Microalgal Growth

**Figure 1:**
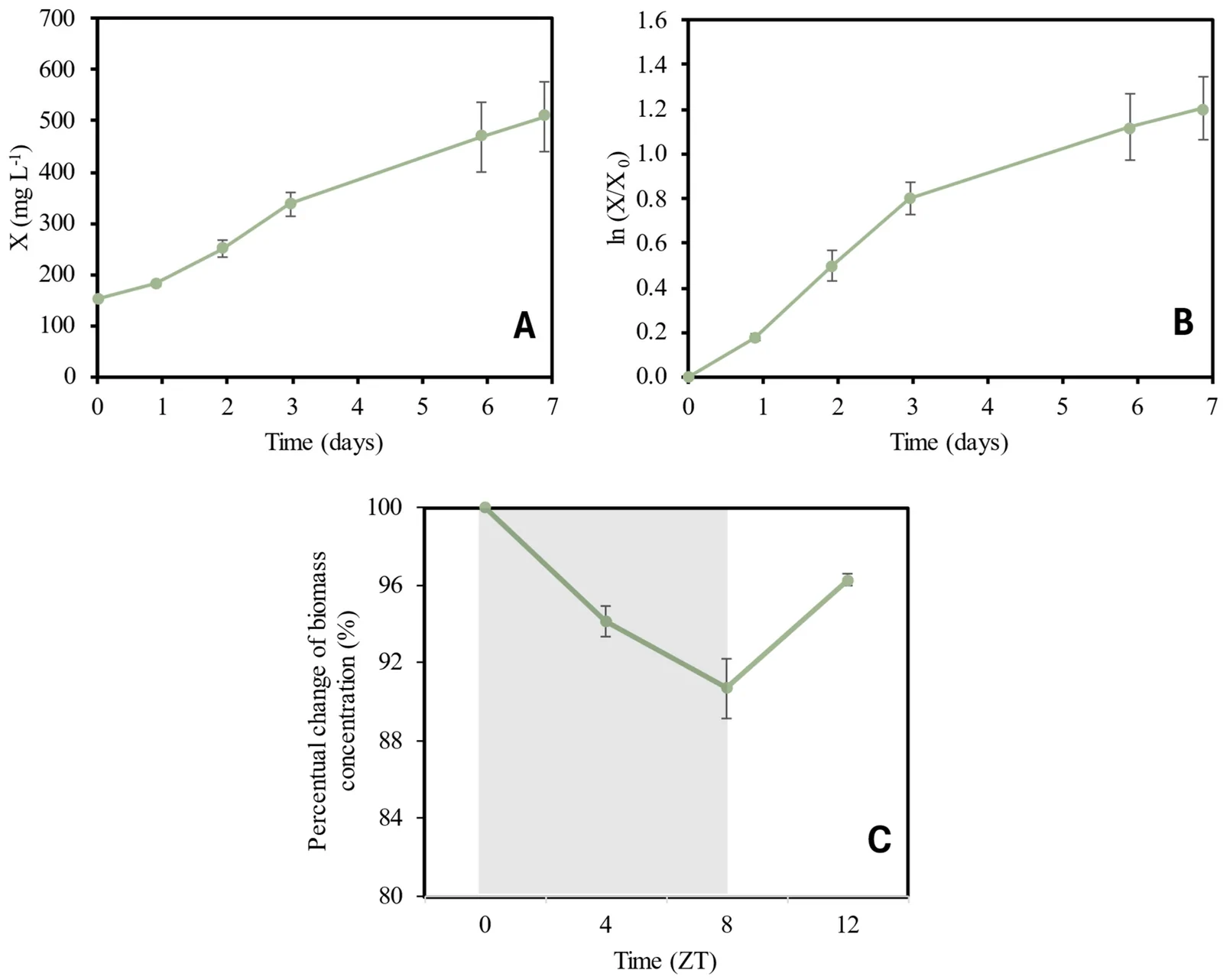
(**A**) Microalgal biomass concentration and (**B**) normalised logarithmic growth curve expressed as ln(X/X_0_), over a seven-day cultivation period. (**C**) Relative biomass concentration changes (%) over a 12 h sampling window (ZT0 to ZT12) on day 7 of cultivation under a 16:8 h photoperiod. Values are normalised to the biomass concentration at the start of day 7. Values were measured in the duplicate samples that underwent the full 12 h sampling window (harvested at ZT12). The grey area in the graph represents the dark phase of the photoperiod.

To monitor *C. vulgaris* growth throughout the experimental period, microalgal biomass concentration was measured daily over the seven days of cultivation and, on the harvesting day, at ZT0, ZT4, ZT8, and ZT12. Figure 1A illustrates the biomass concentration (calculated from the measured OD values through Equation (1)) over the cultivation period (7 days), and Figure 1B shows a growth model (ln(X/X_0_)) over the same cultivation period. Figure 1C illustrates the relative variations in biomass concentration during the night phase of day 7. During the 7-day growth period, a daily increase in microalgal biomass was observed, with the exponential growth phase until day three, while the onset of the stationary phase became evident during days six and seven. The specific growth rate was determined at 0.28 ± 0.03 d^−1^ (calculated using Equation (2), with an exponential phase from days 0 to 3—see Figure 1B), which is within values reported in the literature for similar conditions, typically ranging from 0.20 to 0.35 d^−1^ [8,9,20]. Factors such as light intensity, photoperiod and area-to-volume ratios significantly affect the growth rate of these cultures, explaining the differences between the various studies.

Regarding the relative changes in microalgal biomass concentration on the harvest day for the analysed twelve-hour period, a reduction was observed during the dark period, from 508 mg L^−1^ to 461 mg L^−1^. This corresponds to a reduction of 9 ± 1% over the eight hours of light absence (ZT0 to ZT8). After four hours of light (from ZT8 to ZT12), a recovery of 6 ± 1% in biomass concentration was observed.

The decrease in microalgae biomass concentration during the dark phase of the photoperiod is primarily attributed to nighttime respiration [21]. In the dark, microalgae mobilise stored energy reserves for metabolic processes, including DNA replication and cell division, which are scheduled nocturnally to minimise the risk of DNA damage from ultraviolet radiation [22,23]. Therefore, during the light phase, cells tend to increase in size, and during the night period, they perform cell division [7].

A study by Kato et al. [7] showed that, similarly to the variations in biomass concentrations observed in the present work, the biomass concentration of *Chlamydomonas* sp. JSC4 under a light:dark cycle decreased during the dark period and recovered during the light phase. These fluctuations support the framework in which cell growth is coordinated with the circadian rhythm, with cell growth occurring during the light phase and the consumption of reserve compounds during the dark period [3,24]. The nighttime biomass loss can vary significantly depending on the species, operational variables, and the culture physiological condition, with literature reporting biomass declines exceeding 30% in certain instances [25,26]. For instance, in the cultivation of *Chlorella sorokiniana* under a photoperiod of 16:8 h, Holdmann et al. [27] reported an average night biomass loss between 13.1 and 40.1 mg L^−1^ d^−1^, depending on the cultivation conditions. Additionally, Carneiro et al. [28] reported an average night biomass loss of 7–32% for *Nannochloropsis oceanica*, depending on the night temperature and mean daily irradiance.

### 3.2. General Biochemical Composition

#### 3.2.1. Pigments

**Figure 2:**
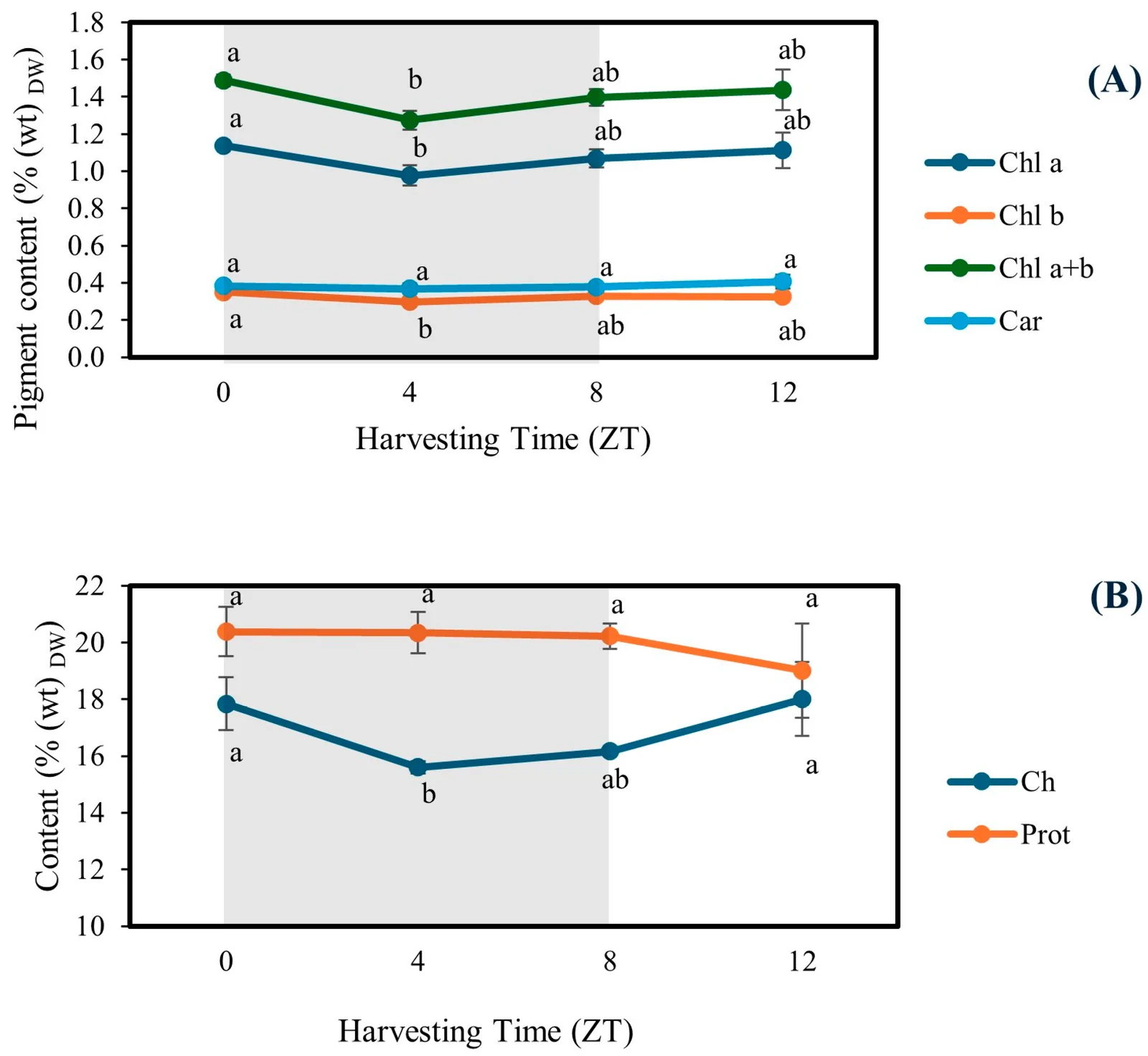
(**A**) Pigments, (**B**) protein and carbohydrate contents variation over a 12 h sampling window (ZT0 to ZT12) on day 7 of cultivation under a 16:8 h photoperiod. The grey area in the graph represents the dark phase of the photoperiod; Chl a—chlorophyll a, Chl b—chlorophyll b, Chl a + b—chlorophyll a + b, Car—Carotenoids, Ch—carbohydrate, Prot—protein.

As can be observed in Figure 2A, the total carotenoid content showed no significant variations throughout the photoperiod, averaging approximately 0.38 ± 0.02% (wt)_DW_ over the 12 h studied window. On the other hand, both chlorophyll a and b decreased during the first four hours of the dark period (ZT0-ZT4), with chlorophyll a declining by 14.1% (from 1.14 ± 0.02% (wt)_DW_ to 0.98 ± 0.05% (wt)_DW_) and chlorophyll b by 15.2% (from 0.35 ± 0.02% (wt)_DW_ to 0.297 ± 0.006% (wt)_DW_). This was followed by a recovery in ZT12, with increases of 13.8% and 9.2% for chlorophyll a and b, respectively. After 4 h in the light period (ZT12), chlorophyll a reached 1.1 ± 0.1% (wt)_DW_ and chlorophyll b 0.33 ± 0.01% (wt)_DW_. A decrease in cell pigment content in the absence of light has been reported in previous studies on *Nannochloropsis* sp., where both chlorophyll and carotenoid contents declined under dark conditions [3,5]. Other studies report the opposite trend, with pigment accumulation during the night period. Hidasi and Belay [6] showed an increase in both chlorophyll and carotenoid contents during the night period in *A. platensis* across three outdoor experiments conducted in consecutive months. This behaviour was suggested to be associated with exposure to high light intensities around midday, which might have induced photoinhibition, particularly in the absence of efficient photoprotective mechanisms such as the xanthophyll cycle [6]. Conversely, the present study provides no evidence of photoinhibition, as chlorophyll content tends to increase during light periods. These differences can also be related to different light intensities (which affects the total quantity of light in a cycle that microalgae are exposed to) and strain-specific metabolism. Esteves et al.’s [20] results also reveal that the strain used in the current study showed no signs of photoinhibition, with increasing specific growth rates from 291 to 1107 μmol m^−2^ s^−1^. Moreover, the observed results suggest that chlorophyll expression largely depends on light availability, whereas overall carotenoid regulation is less affected by the light:dark transition. Furthermore, pigment composition is also governed by other factors such as light quality. For instance, exposure to blue light was associated with a higher chlorophyll content compared to white and red lights in *C. vulgaris* [29].

#### 3.2.2. Protein

Regarding protein content, no statistical variations were registered over the light:dark cycle, as represented in Figure 2B. Similar results were reported by Sui et al. [24] and Cuhel et al. [30], who observed stable protein levels in *Dunaliella* sp. over a 24 h cycle under 12:12 h and 16:8 h photoperiod regimes, respectively. On the other hand, Fábregas et al. [5] and Sukenik and Carmeli [3] reported an increase in protein content during the light phase, followed by a decrease during the night cycle with *Nannochloropsis* sp., a pattern also observed by Ogbonna and Tanaka [10] in *C. pyrenoidosa*. A study conducted by de Winter et al. [4] with *Neochloris oleoabundans* reported increased protein content during the cellular growth phase and decreased protein content during cell division. However, these processes were not always synchronised with the light and dark phases. While working with *A. platensis*, Hidasi and Belay [6] observed stable protein content during the night phase and a further decline towards the end of the light period. This pattern was observed over three consecutive months and was attributed to intensive light exposure in outdoor experiments, which might have influenced protein synthesis dynamics. Overall, the protein trends observed in the present study are consistent with some of the available literature, suggesting that these variations are species-dependent.

#### 3.2.3. Carbohydrates

Carbohydrate levels showed a 12.5% decrease in the night cycle, followed by a full recovery after four hours of light, as illustrated in Figure 2B. The highest values were observed at ZT0 and ZT12, reaching 17.8 ± 0.9 and 18.0 ± 1.3% (wt)_DW_ of biomass, respectively. A similar trend was observed by Kato et al. [7], who reported a decrease in carbohydrate content during the night period. Fábregas et al. [5] and Sukenik and Carmeli [3] recorded similar variation patterns with a continuous carbohydrate increase during the light phase, followed by a decrease in the dark period in *Nannochloropsis*, a pattern also reported by Ogbonna and Tanaka [10] in *C. pyrenoidosa* and Hidasi and Belay [6] in *A. platensis*. Additionally, de Winter et al. [4] described an increase in starch content after 6–7 h after sunrise, followed by a decrease once illumination ceased. In all cases, these carbohydrate fluctuations are attributed to their synthesis during the light phase for subsequent consumption in the dark.

The reduction in intracellular carbohydrate content during the night phase is primarily attributed to its role as a critical energy buffer and carbon storage source. During the light period, microalgae accumulate starch. In contrast, in the absence of light, these reserves are rapidly mobilised to fulfil maintenance energy requirements and sustain essential physiological activities while photosynthesis is inactive [22]. It has been reported that during the dark phase, the pentose phosphate pathway is active in microalgae glucose metabolism, providing energy and precursors [31].

### 3.3. Carotenoid Profile

The carotenoid profile over the twelve hours is detailed in Table 1. Of the pigments identified, neoxanthin, lutein, and β-carotene presented constant concentrations with no significant changes, while violaxanthin and zeaxanthin levels showed complementary fluctuations. During the dark period, between ZT0 and ZT8, violaxanthin levels increased by 27.2% (from 0.38 ± 0.03% (wt)_DW_ to 0.483 ± 0.002% (wt)_DW_) while zeaxanthin content declined by 38% (from 0.32 ± 0.07% (wt)_DW_ to 0.20 ± 0.01% (wt)_DW_). This interconversion is characteristic of the xanthophyll cycle, a coordinated photoprotective response to excessive light in photosynthetic organisms such as plants and microalgae, including *C. vulgaris*. During high light intensity exposure, the violaxanthin cycle is activated, and violaxanthin, a di-epoxy carotenoid, is enzymatically converted into zeaxanthin through the intermediate antheraxanthin to protect the photosynthetic system from light-induced stress. In lower light intensity and darkness, the reverse reaction is favoured, and zeaxanthin is again converted into violaxanthin [32,33,34]. Accordingly, the present data showed an increase in violaxanthin and a corresponding decrease in zeaxanthin during the dark phase, supporting this biochemical scheme. Despite differences among individual carotenoids, the total carotenoid composition is consistent with the general pigment analysis previously reported, which showed no significant differences in total carotenoid levels throughout the light:dark cycle (Figure 2A).

The present study reveals that lutein accounted for 60–70% of the total identified carotenoid content. Similarly, a previous study reported lutein as the predominant carotenoid in *C. vulgaris* [8]. Lutein is a carotenoid with strong antioxidant power that shows anticancer properties and benefits to both eye and cardiovascular health, and can be used as a dietary supplement [35].

### 3.4. Fatty Acid Profile

The fatty acid profile was analysed over the same defined period, thereby identifying eight distinct fatty acids using the method described in the previous section. Table 2 shows the fatty acid profile relative to biomass weight, and Figure S4 (see Supplementary Materials) shows the relative percentage of different fatty acid groups in the analysed samples. Total saturated (SFA), monounsaturated (MUFA), and polyunsaturated fatty acids (PUFA) accounted for, respectively, 17%, 5% and 63% of total fatty acids. For the major fatty acid groups and total fatty acids, no significant differences were identified across the dark phase and beginning of the light phase, even though minor differences were detected for specific fatty acids (not relevant for further applications in biomass valorisation). A similar trend was observed in de Winter et al.’s [4] study, in which the content of total fatty acids remained constant throughout a light:dark cycle in *N. oleoabundans.* In contrast, other studies reported a decrease in lipid content during the night period in *Chlamydomonas* sp. and *Nannochloropsis* sp. [3,5,7]. Such discrepancies in lipid dynamics may be associated with differences in metabolic regulation across the light:dark cycle, including the utilisation of stored lipids as an energy source at night, besides carbohydrates [4,7].

Linoleic acid (LA; C18:2) and α-linolenic acid (ALA; C18:3) are essential fatty acids found in *C. vulgaris* in high proportions, representing over 17% and 52% of total PUFA in the present study, respectively. They play crucial roles as precursors in the biosynthesis of other long-chain ω-3 and ω-6 PUFA involved in key physiological processes [36,37,38]. Since the human body cannot synthesise them, they must be obtained through dietary means or direct supplementation [37,38,39]. Consequently, PUFA-enriched food products and dietary supplements have gained increasing interest as strategies to improve fatty acid intake profiles and support human health [40]. However, care should be taken with respect to the oxidative stability of these products.

### 3.5. Future Directions

Taken together, the variations in biomass concentration and biochemical composition quantified in this study have direct implications for the process design and economic feasibility of *C. vulgaris* biorefineries. Synthesising these multi-parameter dynamics, the end of the light period (ZT0) represented the most advantageous harvesting point. At the end of the dark phase (ZT8), a reduction of 9 ± 1% in biomass concentration (decreasing from 508 mg L^−1^ to 461 mg L^−1^) was observed, alongside a 12.5% depletion of carbohydrate content due to nighttime metabolic respiration. Furthermore, since total proteins and fatty acids remained stable across the analysed 12 h period, their productivity is only related to biomass concentration. Consequently, operating with harvesting times at the end of the light period (ZT0) maximises component productivity in commercial production systems.

Further studies investigating the impact of harvesting time should be conducted at a pilot scale and continuous cultivation strategies to closely mimic industrial settings. Furthermore, considering industrial production and commercial applications, it is fundamental to maximise the content and productivity of target compounds. Therefore, different strategies, including the optimisation of other cultivation conditions, the use of genetic engineering tools, and the adaptive laboratory evolution (ALE) of the strain under specific environmental stressors, should be further evaluated with the aim of large-scale industrial bioprocessing of *C. vulgaris*. There is already a wide range of literature available on these topics. For instance, Pinto et al. [41] evaluated the impact of cultivation temperature, time and N:P ratio in *C. vulgaris*. Results showed that the maximum protein productivity occurred at the highest tested N:P ratio (42.6) and the lowest temperature (18 °C) after 4 days of cultivation. Regarding the ALE of this species, Varunraj et al. [42] performed 35 cycles of cultivation under high salt concentrations, obtaining a strain with increased lipid content and antioxidant capacity, with a minor reduction in growth rate when compared to the control. Furthermore, Trovão et al. [43] obtained an increase in biomass productivity and protein and chlorophyll contents of 38%, 19% and 62%, respectively, after random mutagenesis with fluorescence-activated cell sorting. Also, Lin and Ng [44] used a CRISPR/Cas9 system to genetically modify *C. vulgaris* and obtained a strain with higher lipid accumulation (46% increase). Xie et al. [45] evaluated the use of 3% CO_2_ enriched aeration (compared to atmospheric air) in a pilot-scale (5 m^3^) outdoor open raceway pond, using real swine wastewater for biodiesel production. Results demonstrate that 3% CO_2_ supplementation enhanced both microalgal growth and lipid content. These examples and the results from the current study highlight the potential of the listed strategies for application in industrial microalgae cultivation.

## 4. Conclusions

The present study investigated the influence of circadian rhythms by analysing variations in the growth and biochemical composition of *C. vulgaris* over a 16:8 h photoperiod. Results showed a 9 ± 1% decrease in biomass concentration during the dark phase, along with a reduction of about 12.5% in carbohydrate content, suggesting consumption of stored reserves throughout this period. During the light period, this microalga uses photosynthetic energy to support biomass growth and carbon accumulation, with carbohydrate levels recovering after four hours of illumination and biomass concentration increasing by 6%. Pigment variation has also revealed interesting metabolic dynamics throughout the cycle. Total chlorophyll content decreased by 14.4% during the dark phase and was progressively restored after the onset of light. In addition, complementary fluctuations between violaxanthin and zeaxanthin were observed, providing evidence of an active xanthophyll cycle in *C. vulgaris* as a photoprotective response to light conditions.

These findings demonstrate that light:dark cycling strongly modulates biomass productivity and biochemical partitioning in *C. vulgaris*. Based on the results obtained, the end of the light period represents the optimal operational window for biomass harvesting, ensuring maximum biomass concentration and peak levels of key compounds (e.g., carbohydrates and pigments) prior to nocturnal degradation. Understanding these circadian-dependent metabolic adjustments is crucial for optimising harvesting strategies and enhancing the efficiency of microalgal production systems for biotechnological applications.

## Figures and Tables

**Table 1:** Changes in the profile of carotenoids over a 12 h sampling window (ZT0 to ZT12) on day 7 of cultivation under a 16:8 h photoperiod.

Time (ZT)	Content (mg g_DW_^−1^)				
	Neoxanthin	Violaxanthin	Lutein	Zeaxanthin	β-Carotene
0	0.70 ± 0.08 ^a^	0.38 ± 0.04 ^b^	3.4 ± 0.3 ^a^	0.32 ± 0.07 ^a^	0.33 ± 0.03 ^a^
4	0.61 ± 0.03 ^a^	0.43 ± 0.02 ^b^	3.1 ± 0.1 ^a^	0.24 ± 0.02 ^ab^	0.32 ± 0.01 ^a^
8	0.662 ± 0.003 ^a^	0.483 ± 0.002 ^a^	3.19 ± 0.01 ^a^	0.20 ± 0.01 ^b^	0.30 ± 0.04 ^a^
12	0.651 ± 0.003 ^a^	0.497 ± 0.003 ^a^	3.14 ± 0.03 ^a^	0.21 ± 0.01 ^b^	0.302 ± 0.004 ^a^

Results are expressed as mean ± standard deviation, *n* = 3. Different lowercase letters within a column indicate significant differences (*p* < 0.05).

**Table 2:** Changes in the profile of fatty acids over a 12 h sampling window (ZT0 to ZT12) on day 7 of cultivation under a 16:8 h photoperiod.

Fatty Acid	Time (ZT)			
	0	4	8	12
C16:0	1.66 ± 0.03 ^a^	1.66 ± 0.03 ^a^	1.75 ± 0.01 ^a^	1.73 ± 0.05 ^a^
C16:1	0.165 ± 0.009 ^a^	0.163 ± 0.003 ^ab^	0.146 ± 0.005 ^b^	0.151 ± 0.003 ^ab^
C16:2	0.51 ± 0.03 ^ab^	0.468 ± 0.004 ^b^	0.534 ± 0.008 ^a^	0.49 ± 0.01 ^ab^
C16:3	n.d.	n.d.	0.13 ± 0.01 ^a^	0.12 ± 0.01 ^a^
C16:4	1.07 ± 0.07 ^a^	0.991 ± 0.001 ^ab^	0.94 ± 0.01 ^b^	0.92 ± 0.02 ^b^
C18:1	0.34 ± 0.02 ^a^	0.31 ± 0.02 ^a^	0.361 ± 0.002 ^a^	0.35 ± 0.03 ^a^
C18:2	1.14 ± 0.07 ^ab^	1.081 ± 0.006 ^b^	1.224 ± 0.006 ^a^	1.16 ± 0.03 ^ab^
C18:3	3.6 ± 0.3 ^a^	3.42 ± 0.02 ^a^	3.44 ± 0.02 ^a^	3.38 ± 0.09 ^a^
SFA	1.66 ± 0.03 ^a^	1.66 ± 0.03 ^a^	1.75 ± 0.01 ^a^	1.73 ± 0.05 ^a^
MUFA	0.51 ± 0.03 ^a^	0.47 ± 0.02 ^a^	0.51 ± 0.01 ^a^	0.51 ± 0.02 ^a^
PUFA	6.3 ± 0.4 ^a^	6.3 ± 0.4 ^a^	5.99 ± 0.03 ^a^	6.27 ± 0.04 ^a^
Others	1.50 ± 0.07 ^a^	1.42 ± 0.09 ^a^	1.43 ± 0.01 ^a^	1.36 ± 0.05 ^a^
Total	10.1 ± 0.6 ^a^	9.5 ± 0.1 ^a^	9.96 ± 0.05 ^a^	9.7 ± 0.3 ^a^

Results are expressed as mean ± standard deviation, *n* = 3, expressed in content of fatty acid relative to biomass weight (% (wt)_DW_). Different lowercase letters within a row indicate significant differences (*p* < 0.05). n.d.—Value below detection limit; non-identified fatty acids and identified fatty acids whose relative percentage relative to total fatty acid weight is below 1% are included in line “Others”. SFA, MUFA and PUFA correspond to saturated, monounsaturated and polyunsaturated fatty acids.

## Data Availability

The original contributions presented in this study are included in the article and Supplementary Material. Further inquiries can be directed to the corresponding author.

## Supplementary Materials

The following supporting information can be downloaded at https://www.mdpi.com/article/10.3390/biotech15030064/s1; Table S1: Detailed composition of modified OECD medium; Figure S1: Calibration curve relating optical density (OD) at 680 nm to dry biomass concentration (X) of *Chlorella vulgaris*; Figure S2: BSA protein standard calibration curves for low (750 nm)- and high (500 nm)-concentration working ranges; Figure S3: Glucose standard calibration curve (490 nm); Figure S4: Relative percentage of different fatty acid groups (SFA—Saturated fatty acids; MUFA—Monounsaturated fatty acids; PUFA—Polyunsaturated fatty acids) over a 12 h sampling window (ZT0 to ZT12) on day 7 of cultivation under a 16:8 h photoperiod. Non-identified fatty acids and identified fatty acids whose relative percentage relative to total fatty acid weight is below 1.0% are included in “Others”. Error bars correspond to standard deviation (*n* = 3). Different lowercase letters in bars of the same colour indicate significant differences (*p* < 0.05).
