# Supplementary material for: Night-Time Biomass and Compositional Dynamics in Chlorella vulgaris: Optimisation of Harvesting Time
Source: BioTech (Basel). 2026 Aug 6;15(3):64. doi: 10.3390/biotech15030064 (PMC13510351; doi:10.3390/biotech15030064)
Supplement: Supplementary file 1 [file biotech-15-00064-s001.zip › biotech-4452648-supplementary.pdf]

BioTech MDPI

Supplementary Materials: Night-time biomass and compositional dynamics in *Chlorella vulgaris*: Optimisation of harvesting time

Sofia Pires, Susana Casal, Tânia G. Tavares, José C. M. Pires and Joana Oliveira

The composition of the modified OECD medium and the supplier of each used compound are detailed in Table S1.

Table S1. Detailed composition of the modified OECD medium.

Reagent	Supplier	Content in medium (mg L^{-1})
$NaNO_3$	Merck KGaA (Darmstadt, Germany)	250
$MgCl_2·6H_2O$	VWR (Radnor, Pennsylvania, United States)	12
$CaCl_2·2H_2O$	VWR	18
$MgSO_4·7H_2O$	VWR	15
KH_2PO_4	VWR	45
$FeCl_3·6H_2O$	Merck KGaA	0.08
$Na_2EDTA·2H_2O$	VWR	0.1
H_3BO_3	VWR	0.185
$MnCl_2·4H_2O$	VWR	0.415
$ZnCl_2$	VWR	$3×10^{-3}$
$CoCl_2·6H_2O$	TCI (Tokyo, Japan)	$1.5×10^{-3}$
$CuCl_2·2H_2O$	VWR	$0.01×10^{-3}$
$Na_2MoO_4·2H_2O$	VWR	$7×10^{-3}$
$NaHCO_3$	VWR	500

The calibration curves for biomass, protein and carbohydrate concentrations are presented in Figures S1, S2 and S3, respectively.

https://doi.org/10.3390/biotech15030064

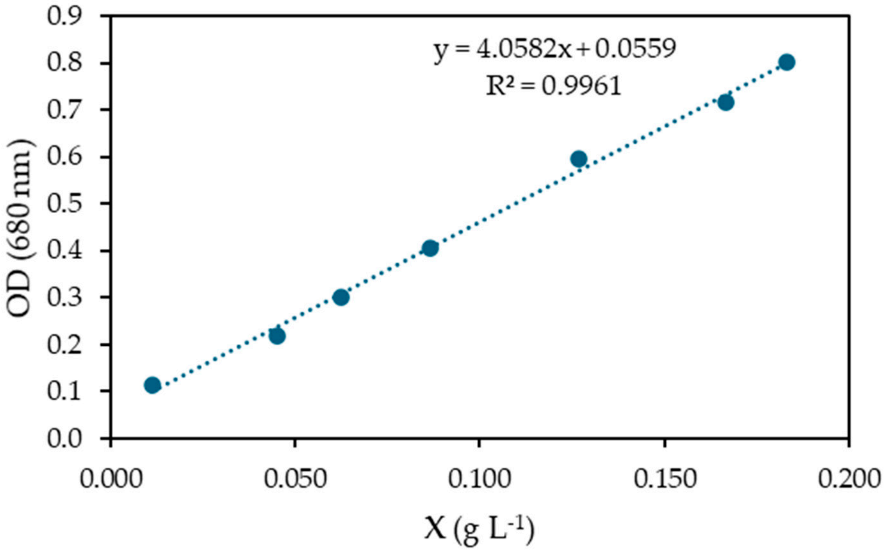

Figure S1. Calibration curve relating optical density (OD) at 680 nm to dry biomass concentration (X) of *Chlorella vulgaris*.

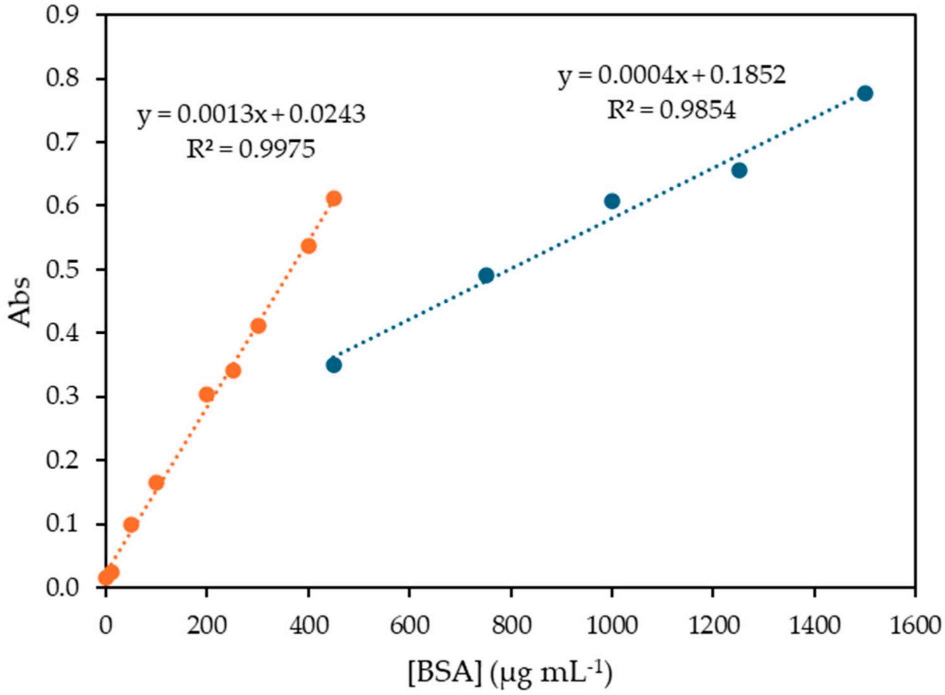

Figure S2. BSA protein standard calibration curves for low (750 nm)- and high (500 nm)-concentration working ranges.

https://doi.org/10.3390/biotech15030064

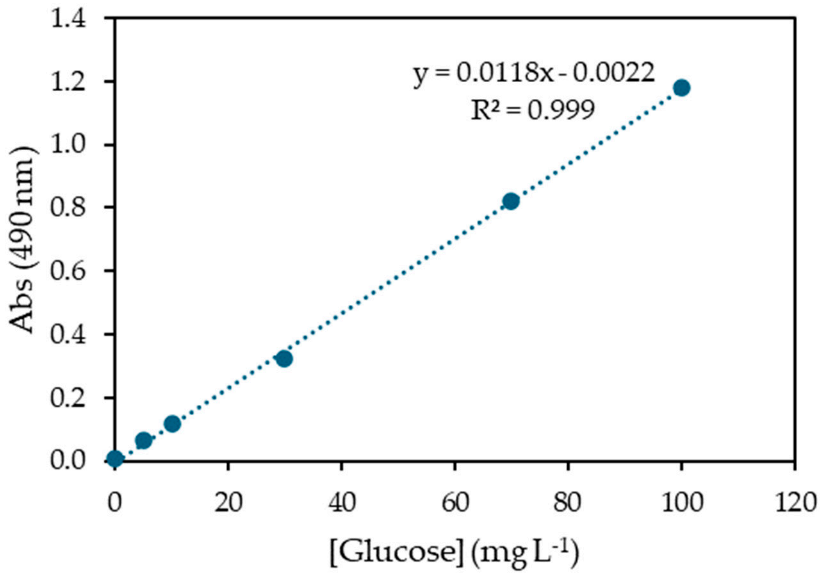

Figure S3. Glucose standard calibration curve (490 nm).

Figure S4 shows the relative percentage of different fatty acid groups over a 12 hour sampling window (ZT0 to ZT12) on day 7 of cultivation under a 16:8 h photoperiod.

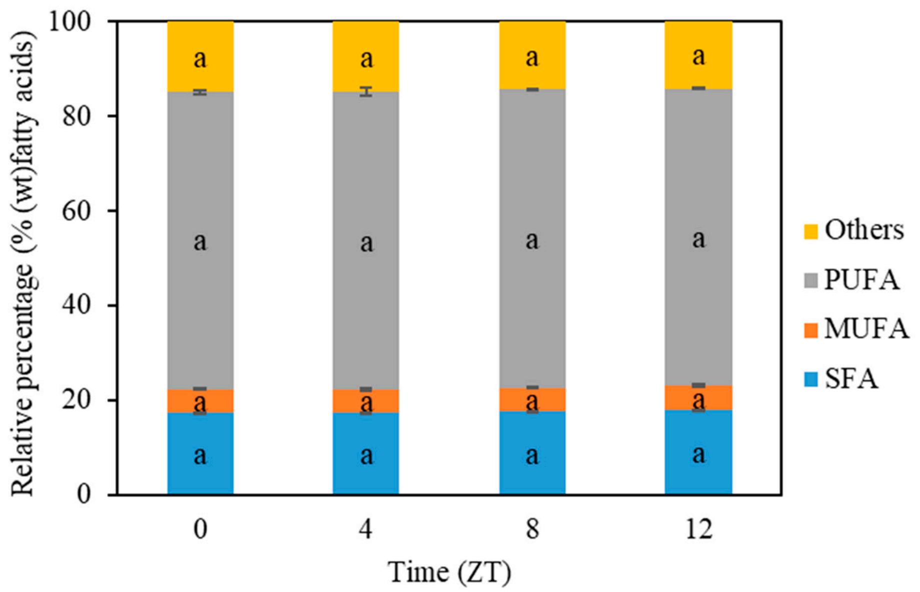

Figure S4. Relative percentage of different fatty acid groups (SFA – saturated fatty acids; MUFA – monounsaturated fatty acids; PUFA – polyunsaturated fatty acids) over a 12 hour sampling window (ZT0 to ZT12) on day 7 of cultivation under a 16:8 h photoperiod. Non-identified fatty acids and identified fatty acids whose relative percentage relative to total fatty acid weight is below 1.0 % are included in "Others". Error bars correspond to standard deviation (n=3). Different lowercase letters in bars of the same color indicate significant differences ($p < 0.05$) .
